# Dendritic and Axonal Propagation Delays May Shape Neuronal Networks With Plastic Synapses

**DOI:** 10.3389/fphys.2018.01849

**Published:** 2018-12-20

**Authors:** Mojtaba Madadi Asl, Alireza Valizadeh, Peter A. Tass

**Affiliations:** ^1^Department of Physics, Institute for Advanced Studies in Basic Sciences (IASBS), Zanjan, Iran; ^2^School of Cognitive Sciences, Institute for Research in Fundamental Sciences (IPM), Tehran, Iran; ^3^Department of Neurosurgery, Stanford University School of Medicine, Stanford, CA, United States

**Keywords:** propagation delays, spike-timing-dependent plasticity, synchronization, mathematical modeling, living systems

## Abstract

Biological neuronal networks are highly adaptive and plastic. For instance, spike-timing-dependent plasticity (STDP) is a core mechanism which adapts the synaptic strengths based on the relative timing of pre- and postsynaptic spikes. In various fields of physiology, time delays cause a plethora of biologically relevant dynamical phenomena. However, time delays increase the complexity of model systems together with the computational and theoretical analysis burden. Accordingly, in computational neuronal network studies propagation delays were often neglected. As a downside, a classic STDP rule in oscillatory neurons without propagation delays is unable to give rise to bidirectional synaptic couplings, i.e., loops or uncoupled states. This is at variance with basic experimental results. In this mini review, we focus on recent theoretical studies focusing on how things change in the presence of propagation delays. Realistic propagation delays may lead to the emergence of neuronal activity and synaptic connectivity patterns, which cannot be captured by classic STDP models. In fact, propagation delays determine the inventory of attractor states and shape their basins of attractions. The results reviewed here enable to overcome fundamental discrepancies between theory and experiments. Furthermore, these findings are relevant for the development of therapeutic brain stimulation techniques aiming at shifting the diseased brain to more favorable attractor states.

## 1. Introduction

Time delays play an important role in various fields of physiology (Glass et al., 1988; Batzel and Kappel, 2011). Neurophysiological time delays crucially affect generation, transmission, and processing of information among different components of a living system, and more specifically, between interconnected neurons in the nervous system. The time required for neuronal communication can be significantly prolonged due to the physical distance between sending and receiving units (Knoblauch and Sommer, 2003, 2004), finite velocity of signal transmission (Desmedt and Cheron, 1980), morphology of dendrites and axons (Manor et al., 1991; Boudkkazi et al., 2007) and information processing time of the cell (Wang et al., 2009). The physiological range of such time delays may vary from a few milliseconds in dendritic trees (Agmon-Snir and Segev, 1993; Schierwagen and Claus, 2001) to tens of milliseconds in axonal components of cortico-thalamic circuits (Swadlow and Weyand, 1987).

The presence of such experimentally observed propagation delays can have significant impacts on the performance, structure, and function of the nervous system (Sirota et al., 2005; Joris and Yin, 2007; Chomiak et al., 2008; Spencer et al., 2012, 2018; Squire et al., 2012; Walters et al., 2013; Esfahani et al., 2016; Stoelzel et al., 2017). In fact, the diversity of dendritic and axonal propagation delays in the nervous system can underlie different response properties of the relevant neuronal populations (Sirota et al., 2005; Stoelzel et al., 2017). For instance, axonal propagation delays in visual and motor cortico-thalamic circuits correspond to different response functions associated with sensory, movement-related, or spontaneous activity of neurons (Sirota et al., 2005; Stoelzel et al., 2017). The auditory system employs compensatory delay mechanisms to modulate the asynchrony in inputs, in this way reducing the sensitivity of brainstem neurons to interaural time delays (Spencer et al., 2012, 2018). Propagation delays also can affect the communication between connected neurons by modulating the spatio-temporal properties of pre- and postsynaptic activity patterns (Chomiak et al., 2008). One major role of axonal propagation delays might be their involvement in the generation of nearly synchronous responses in postsynaptic neurons by regulating the outgoing impulses in axons with several postsynaptic target neurons (Chomiak et al., 2008).

Despite their inevitable physiological significance in living systems, propagation delays are usually overlooked in mathematical models, presumably to avoid further complexity. Although this assumption simplifies the theoretical calculations and reduces the computational cost of multiscale computer simulations, it renders mathematical models unable to provide insight into relevant physiological mechanisms. However, a number of theoretical and computational studies have shown that propagation delays modify weight and neuronal dynamics by affecting the co-evolution of synaptic strengths and neuronal activity, and therefore, shaping the emergent functional and structural properties of plastic neuronal networks (Lubenov and Siapas, 2008; Aoki and Aoyagi, 2009; Kozloski and Cecchi, 2010; Rubinov et al., 2011; Knoblauch et al., 2012; Babadi and Abbott, 2013; Kerr et al., 2013; Madadi Asl et al., 2017, 2018a), where the synaptic strengths are regulated by spike-timing-dependent plasticity (STDP) (Gerstner et al., 1996; Markram et al., 1997; Bi and Poo, 1998; Song et al., 2000). Hence, incorporation of time delays in mathematical models can significantly modify the dynamical properties of neuronal systems, such as the emergence of different connectivity patterns (Madadi Asl et al., 2017, 2018a), affecting the dynamics of fixed points and synchronization properties between interconnected neurons (D'Huys et al., 2008; Popovych et al., 2011), and the emergence of different multistable dynamical attractors (Song et al., 2009; Madadi Asl et al., 2018a).

Neglecting realistic time delays in mathematical models has led to discrepancies between theoretical and experimental findings over the past few years. In this manuscript, we review recent physiological and computational studies that have shown that a simple classic STDP rule enhanced by realistic dendritic and axonal propagation delays is able to explain some of the corresponding experimental results. We highlight the pivotal role of dendritic and axonal propagation delays in regulating the emergent activity and connectivity patterns in plastic neuronal networks under the influence of classic pair-based STDP which significantly affects the information transmission in neuronal populations. Ultimately, we point out the importance of propagation delays in the computation-based development of therapeutic brain stimulation techniques that are used for modulating plastic neuronal networks in diseased brains.

## 2. Propagation Delays: Physiological Aspects

From a physiological standpoint, the measurement of propagation delays in dendrites or axons of neuronal populations requires complex experimental setups, stimulation protocols, or modern clinical instruments. Several experimental studies investigated dendritic and axonal propagation delays in neuronal populations of various species (Swadlow and Weyand, 1987; Swadlow, 1990; Agmon-Snir and Segev, 1993; Schierwagen and Claus, 2001; Ferraina et al., 2002; Briggs and Usrey, 2009; Stoelzel et al., 2017). The physiological range of dendritic and axonal propagation delays may attain a range of different values, based on different experimental model systems in which they were measured. For instance, the value of dendritic propagation delays may vary from sub-milliseconds to a few milliseconds (Agmon-Snir and Segev, 1993; Schierwagen and Claus, 2001). Axonal propagation delays, however, may take a wider range from a few milliseconds in cortico-tectal connections (Swadlow and Weyand, 1987) to tens of milliseconds in cortico-cortical (Swadlow, 1990) and cortico-thalamic circuits (Swadlow and Weyand, 1987). Axonal delays are typically greater than dendritic delays in a neuron, however, values of dendritic delays greater than the axonal delays were experimentally measured in distal dendrites of neocortical pyramidal neurons (Stuart and Spruston, 1998; Sjöström and Häusser, 2006).

In the auditory system, dendritic and axonal propagation delays modify the mechanisms of interaural time sensitivity by regulating coincident or lagged inputs from the two sides, and therefore, play a constructive/destructive role in binaural sound localization depending on the location of the sound source and the leading ear (Joris and Yin, 2007; Squire et al., 2012). Dendritic propagation delays are hypothesized to play a compensatory role for the input asynchrony in the auditory brainstem of mammals using plastic synaptic weights (Spencer et al., 2012, 2018). In the motor system, propagation delays can impose functional limitations on the efficiency of feedback control in situations where time-critical performance of the sensory feedback is vital for the biological system (Squire et al., 2012). The functional significance of diverse range of axonal propagation delays in cortico-thalamic circuits are shown to be strongly related to multiple visual response properties (Stoelzel et al., 2017). Axonal delays act as a timing mechanism in the neuronal networks responsible for path integration of head direction and were computationally shown to promote the accuracy of path integration in the absence of visual input (Walters et al., 2013). Experimentally delayed visual feedback was used as a tool to manipulate and disentangle different motor control regulatory brain mechanisms (Tass et al., 1996; Rougier, 2003; van den Heuvel et al., 2009).

The role of dendritic or axonal propagation delays has been implicated in a number of nervous system disorders such as Parkinson's disease (PD) (Hauptmann and Tass, 2007; Ebert et al., 2014; Shouno et al., 2017), epilepsy (Wendling et al., 2010), and multiple sclerosis (MS) (Waxman, 2006). Subthalamic nucleus (STN) parkinsonian oscillations are shown to be sensitive to feedback oscillatory inputs of cortical circuits in a delay-dependent manner (Shouno et al., 2017). Neurophysiological latencies are hypothetically involved in the complex propagation mechanisms of epileptic activity in the brain (Wendling et al., 2010). In MS patients a demyelination of axonal components may lead to significant transmission delays along the axon of the cell (Waxman, 2006). This process reduces the conduction velocity of signals along the axon and can ultimately result in a blockage of information transmission and conduction failure of the axon (Waxman, 2006). Furthermore, propagation delays can have significant impact on methods used to record or modulate brain activity. For instance, time delays can affect procedures that estimate the degree of association and phase relationships between electroencephalogram (EEG) signals (Lopes da Silva F et al., 1989), or adjust the performance of therapeutic brain stimulation techniques (see below).

## 3. Propagation Delays: Computational Aspects

From a computational standpoint, one of the most important roles of propagation delays might be their potential to address the challenging inconsistencies between theoretical and computational studies regarding the functional, structural, and dynamical properties of plastic neuronal networks driven by the pair-based STDP (Abbott and Nelson, 2000; Song and Abbott, 2001; Pfister and Gerstner, 2006; Masuda and Kori, 2007; Lubenov and Siapas, 2008; Clopath et al., 2010; Kozloski and Cecchi, 2010; Knoblauch et al., 2012) on the one hand and relevant experimental observations (Bi and Poo, 1998; Van Rossum et al., 2000; Sjöström et al., 2001; Song et al., 2005; Wang et al., 2005; Lea-Carnall et al., 2017) on the other hand, e.g., the prevalence of strong bidirectional loops between pairs of neurons in cortical circuits (Song et al., 2005; Morishima and Kawaguchi, 2006) and the dependence of emergent synaptic structures on the firing rate of neurons (Sjöström et al., 2001; Wang et al., 2005; Lea-Carnall et al., 2017).

In fact, the classic pair-based STDP model (Gerstner et al., 1996; Markram et al., 1997; Bi and Poo, 1998; Song et al., 2000), through which the change of the synaptic strengths is induced by pairwise temporal interactions between pre- and postsynaptic spikes, has shown to be unable to account for the emergence of strong bidirectional connections and neuronal loops (Abbott and Nelson, 2000; Song and Abbott, 2001; Lubenov and Siapas, 2008; Kozloski and Cecchi, 2010; Knoblauch et al., 2012; Babadi and Abbott, 2013). Furthermore, it fails to address the experimentally measured dependency of weight dynamics on the frequency of oscillations (Sjöström et al., 2001; Wang et al., 2005; Lea-Carnall et al., 2017). Several attempts were made in order to overcome the limitations of the pair-based STDP model over the past few years via the introduction of variations or improvements of the STDP model, such as the triplet-based STDP (Pfister and Gerstner, 2006), STDP with shifted learning window (Babadi and Abbott, 2013), or application of independent noise (Popovych et al., 2013; Lücken et al., 2016). Furthermore, there are several biophysical models that attempt to identify variables with specific biophysical quantities and include them in biophysics-based models of STDP (Castellani et al., 2001; Shouval et al., 2002a,b, 2010; Abarbanel et al., 2003; Rachmuth et al., 2011). For instance, Shouval et al. developed a model of long-term potentiation/depression that includes the back propagating potential in the STDP model (Castellani et al., 2001; Shouval et al., 2002a,b). For a review of the shortcomings of pair-based STDP and its variations see (Morrison et al., 2008; Madadi Asl et al., 2018b).

A number of studies, however, have focused on the role of propagation delays to resolve the aforementioned discrepancies. Short axonal propagation delays were shown to decouple synchronous neurons in the presence of STDP (Knoblauch and Sommer, 2003, 2004), whereas long axonal propagation delays promote inter-areal synchronized activity and result in a potentiation of the synaptic strengths (Knoblauch and Sommer, 2004). Taking into account only dendritic propagation delays in the modeling can result in the emergence of strong two-neuron loops (Morrison et al., 2007). Also, it was shown that a combination of dendritic and axonal propagation delays along with an unbalanced STDP profile can lead to the emergence of self-organized states in recurrent neuronal networks (Lubenov and Siapas, 2008). The role of dendritic and axonal propagation delays on the dynamics of recurrent neuronal networks has also been pointed out by considering the effect of time delays in terms of a shift in the STDP temporal window (Babadi and Abbott, 2013). Pairwise interactions of STDP-driven recurrent neuronal populations with such shifts can explain mechanisms underlying loop formation and elimination in bidirectional synapses (Kozloski and Cecchi, 2010; Babadi and Abbott, 2013).

Recently, by presenting a theoretical framework comprising regular spiking neurons we showed that by taking into account dendritic and axonal propagation delays in the modeling of a STDP-driven two-neuron motif different patterns of synaptic connectivity may emerge (Madadi Asl et al., 2017). The synaptic strengths are modified according to the following pair-based STDP rule (Bi and Poo, 1998):

(1) $$\begin{array}{r} \Delta g_{ij}=A_{\pm }\text{sgn}\left(\Delta t^{\prime }\right)\text{exp}\left(-|\Delta t^{\prime }|/\tau_{\pm }\right), \end{array}$$

where *A*_+_(*A*_−_) and τ_+_(τ_−_) are the learning rate and the effective time window of synaptic potentiation (depression), respectively, and sgn(Δ*t*′) is the sign function. Δ*t*′ = Δ*t*+ξ is the effective delayed time lag between pre- and postsynaptic spikes at the synaptic site (Madadi Asl et al., 2017, 2018a). Δ*t* = *t*_post_−*t*_pre_ is the original time lag between pre- and postsynaptic spike pairs, and ξ = τ_d_ − τ _a_ is the difference between dendritic and axonal propagation delays. The synaptic strengths are updated by an additive rule at each step *g*_*ij*_→*g*_*ij*_+Δ*g*_*ij*_, and they are confined in the range (*g*_min_, *g*_max_) ∈ [0, 1] by using a hard bound saturation constraint.

**Figure 1:**
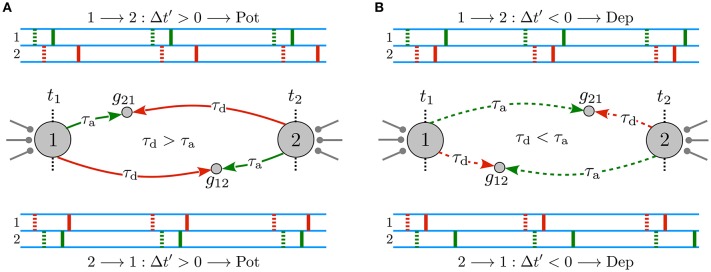
Delay-induced connectivity patterns in a two-neuron motif. Spiking neurons are connected to each other via initially symmetric synapses with strengths *g*_21_ (*g*_12_) of the synapse 1 → 2 (2 → 1) with a small time lag Δ*t* = *t*_post_ − *t*_pre_. Δ*t*′ = Δ*t* + ξ is the effective delayed time lag perceived at the synapse which STDP employs to modify the synapse, where ξ = τ_d_ − τ _a_ and |ξ| > |Δ*t*|. Green and red dotted (solid) markers indicate the original, *t*_1_ and *t*_2_ (delayed) forward and backpropagated spike time of pre- and postsynaptic neurons at the synapse, respectively. **(A)** Emergence of a strong bidirectional loop: both synapses are reciprocally potentiated when τ_d_ > τ _a_. **(B)** A loosely connected motif: both reciprocal synapses are depressed when τ_d_ < τ_a_. Figure partly adopted from Madadi Asl et al. (2018b) with authors' permission.

When propagation delays are ignored or, equivalently, when dendritic and axonal delays are identical for both directions of the reciprocal synapses, ξ = τ_d_ − τ _a_ = 0, the original and the effective delayed time lags are equal, Δ*t*′ = Δ*t*. Therefore, the type of synaptic modification is simply determined by the sign of the original time lag, i.e., Δ*t*≥0 leads to a potentiation of the synapse whereas Δ*t* < 0 results in a depression. Hence, in the absence of propagation delays, the potentiation of one synapse is accompanied by the depression of the other synapse, leading to a unidirectional connection when the potentiation and depression amplitude of the STDP profile is balanced. However, in the presence of dendritic and axonal propagation delays and assuming that the spiking neurons are relatively phase-locked with a small time lag with respect to the propagation delays, |ξ| > |Δ*t*|, the effective delayed time lag Δ*t*′ perceived at the synaptic site may be different from the time lag of the spikes at the cell bodies. Hence, as shown in Figure 1A, when the dendritic delay is greater than the axonal τ_d_>τ_a_, reciprocal synapses are both potentiated, which lead to the emergence of a strong bidirectional loop. On the contrary, greater axonal delays τ_d_ < τ_a_ result in a depression of both reciprocal synapses, in this way generating a loosely connected motif (see Figure 1B).

**Figure 2:**
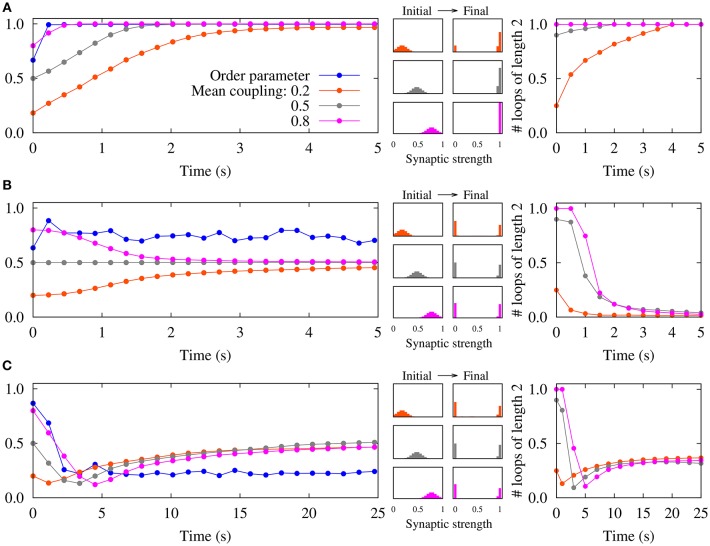
Emergence of different connectivity patterns in a recurrent network of spiking neurons mediated by STDP. (Left panels) Simulated order parameter and time course of three different mean couplings of weight distributions. (Middle panels) Initial Gaussian distribution around different mean values and final distribution of the synaptic strengths. (Right panels) Time course of the normalized number of closed loops of length 2 measuring the number of two-neuron loops in the network (Madadi Asl et al., 2017). **(A)** The synaptic strengths are potentiated and bidirectional connections are significantly enhanced in the inphase firing when τ_d_ = 0.5ms>τ_a_ = 0.3ms. **(B)** STDP breaks strong two-neuron loops and results in unidirectional connections in nearly inphase firing when τ_d_ = τ_a_ = 0.5ms. **(C)** A loosely connected network is achieved where bidirectional loops are eliminated in the nearly antiphase firing when τ_d_ = 0.5ms < τ_a_ = 1.0ms. STDP parameters are *A*_+_ = *A*_−_ = 0.005, and τ_+_ = τ_−_ = 10ms. Figure partly adopted from Madadi Asl et al. (2017) with authors' permission.

By assuming that the neurons remain phase-locked, it was illustrated that the two-neuron results can be extended to recurrent networks of spiking neurons (Madadi Asl et al., 2017, 2018a). Different combinations of dendritic and axonal propagation delays can lead to the emergence of symmetric connections, i.e., either two-neuron bidirectional loops, in the case that dendritic propagation delays are greater than the axonal delays (Figure 2A), or loosely connected motifs when axonal propagation delays are greater than the dendritic delays (Figure 2C) (Madadi Asl et al., 2017). As shown in Figure 2C, the disconnected network is highly unstable and ultimately leads to the emergence of unidirectional connections. However, we showed that the loosely connected motif can be stabilized by assigning a finite value to the lower bound of the synaptic strengths *g*_min_ (Madadi Asl et al., 2018a). In this framework, unidirectional connections can also arise when dendritic and axonal propagation delays are identical in both directions of the reciprocal synapses (Figure 2B).

Furthermore, we studied the emergence of delay-induced multistable dynamics in recurrent networks of spiking neurons attributed to the distribution of the initial synaptic strengths modified by STDP (Madadi Asl et al., 2018a). Such a multistability of the network evolution can be theoretically addressed by the emergence of different attractor states representing the two-dimensional space of the initial synaptic strengths in a two-neuron motif (Madadi Asl et al., 2018a). Moreover, it was shown that the basin of attraction of each dynamical state depends on the firing rate of the neurons in a way that higher firing frequencies favor the emergence of symmetric connections in expense of eliminating the unidirectional connections (Madadi Asl et al., 2018a). Intriguingly, the aforementioned nontrivial dynamics are only present when the dendritic and axonal propagation delays are considered in the neuronal networks models. In the simplest setting, characterized by ignoring dendritic and axonal propagation delays as well as the absence of independent noise, any initial preparation leads to the emergence of unidirectional connections regardless of the neuronal firing pattern and the initial synaptic strengths.

## 4. Concluding Remarks

Propagation delays are inevitable in living systems, and in particular, in the nervous system. The presence of propagation delays has significant impact on the performance, structure, and function of the nervous system. However, from a physiological as well as theoretical standpoint, systems with time delays are considerably more complex, and therefore, delays have typically not been taken into account in relevant studies to simplify the experimental setups in physiological measurements or the mathematical approach in theoretical studies. Incorporating time delays can impose significant levels of complexity and computational cost to the problem. Time delay differential equations are more complicated to deal with from an analytical standpoint. For this reason, in a first approximation, theoretical and computational studies typically ignored the effects of time delays in the modeling. This has led to some discrepancies between theoretical and computational studies with physiological measurements over the past few years (Bi and Poo, 1998; Abbott and Nelson, 2000; Van Rossum et al., 2000; Sjöström et al., 2001; Song and Abbott, 2001; Song et al., 2005; Wang et al., 2005; Pfister and Gerstner, 2006; Masuda and Kori, 2007; Lubenov and Siapas, 2008; Clopath et al., 2010; Kozloski and Cecchi, 2010; Lea-Carnall et al., 2017). However, in an attempt to overcome unphysiological simplifications, we recently demonstrated that incorporating dendritic and axonal propagation delays in STDP-driven networks of spiking model neurons can lead to the emergence of different synaptic connectivity patterns characterized by different dynamical attractors (Madadi Asl et al., 2017, 2018a).

The shortcomings of the pair-based STDP model can be resolved by several improvements proposed during the past decade: The experimentally demonstrated dependency of the weight dynamics on the frequency of the neuronal oscillations can be addressed by considering triplets of spikes (Pfister and Gerstner, 2006) or postsynaptic voltage (Clopath et al., 2010) in the plasticity model. In fact, the triplet-based STDP model is proposed to comply with the experimentally observed dependence of the weight changes on the firing frequency of the oscillations (Sjöström et al., 2001; Wang et al., 2005; Lea-Carnall et al., 2017), showing that bidirectional connections can be promoted at high firing rates (Pfister and Gerstner, 2006). Strong bidirectional loops can be retained by employing an unbalanced STDP model with a shifted learning window (Babadi and Abbott, 2013) or the application of independent noise (Popovych et al., 2013; Lücken et al., 2016). A pair-based STDP model with a rightward shifted learning window was shown to preserve bidirectional connections, provided that potentiation dominates over depression (Babadi and Abbott, 2013). Furthermore, STDP-driven neuronal populations subjected to independent noise counteract the desynchronizing effect of noise by reorganizing their synaptic connectivity (Popovych et al., 2013; Lücken et al., 2016). This ultimately leads to a self-organized noise resistance and promotes bidirectional connections between neurons.

The findings reviewed in this paper highlight the key role of the presence and the range of dendritic and axonal propagation delays in modifying the arising dynamics of synaptic connectivity patterns in recurrent networks of spiking neurons. In fact, short-range propagation delays may favor strong two-neuron loops, whereas connections with long propagation delays may result in the stabilization of a loosely connected network. Hence, the difference of dendritic and axonal propagation delays play a crucial role in determining the final stable coupling regime selected by the network dynamics (Madadi Asl et al., 2017, 2018a). In this way, delay-induced dynamics can overcome the shortcomings of the pair-based STDP model: Strong two-neuron loops can be preserved even with a balanced STDP profile in the absence of independent noise, provided dendritic and axonal propagation delays are considered in the model, and furthermore, the experimentally observed dependency of the weight dynamics on the frequency of the oscillations can be addressed in this setting (Madadi Asl et al., 2017, 2018a).

Abnormal neuronal synchronization is a hallmark of several brain disorders (Lenz et al., 1994; Nini et al., 1995; Hammond et al., 2007). Coordinated reset (CR) stimulation is a computationally developed patterned multichannel stimulation (Tass, 2003) which aims at specifically counteracting abnormal synchrony by desynchronization (Tass, 2003), thereby causing a decrease of neuronal coincidences and, hence, a down-regulation of synaptic weights, ultimately shifting the affected neuronal networks from pathological attractor states (with strong synchrony and strong synaptic connectivity) to more physiological attractor states (with loose coupling and desynchronized activity) (Tass and Majtanik, 2006). The very goal of this approach is to induce long-lasting desynchronization which persists after cessation of stimulation (Tass and Majtanik, 2006). Computationally predicted desynchronizing effects (Tass, 2003), cumulative effects (Hauptmann and Tass, 2009) and long-lasting effects (Tass and Majtanik, 2006) were experimentally validated in the field of deep brain stimulation for the treatment of Parkinson's disease in pre-clinical studies in Parkinsonian monkeys (Tass et al., 2012b; Wang et al., 2016) as well as in a proof of concept study in patients with Parkinson's disease (Adamchic et al., 2014a). As computationally predicted (Popovych and Tass, 2012; Tass and Popovych, 2012), CR stimulation can also be realized by sensory stimulation modalities. Acoustic CR stimulation caused a significant relief of symptoms in patients with chronic subjective tinnitus (Tass et al., 2012a), combined with a significant reduction of abnormal neuronal synchrony (Tass et al., 2012a; Adamchic et al., 2014b) and abnormal effective connectivity (Silchenko et al., 2013), as shown in a proof of concept study employing clinical scores and EEG recordings. By the same token, vibrotactile CR stimulation (Tass, 2017) caused long-lasting treatment effects, as observed in a first in man study in Parkinson's patients (Syrkin-Nikolau et al., 2018).

The findings reviewed above are relevant for the development of desynchronizing brain stimulation techniques. From a model perspective, long-lasting treatment effects are caused by shifting networks from pathological, strongly synchronized model attractor states to physiological, desynchronized attractor states (Tass and Majtanik, 2006). One the one hand, propagation delays determine which attractors actually emerge. On the other hand, propagation delays additionally shape the basins of attraction and, hence, determine to which extent attractors get accessible by appropriate stimulus protocols. Finally, propagation delays may favorably or unfavorably impact on multichannel stimulation protocols with dedicated stimulus sequences, since delays may counteract proper stimulus timing.

## Data Availability Statement

All datasets generated or analyzed for this study are included in the manuscript.

## Author Contributions

AV and PT conceived the study. MM conducted the numerical simulations and theoretical approximations. MM, AV, and PT analyzed the results. MM, AV, and PT wrote and reviewed the paper.

### Conflict of Interest Statement

The authors declare that the research was conducted in the absence of any commercial or financial relationships that could be construed as a potential conflict of interest.
